# Reverse triggering induced by endotracheal tube leak in lightly sedated ARDS patient

**DOI:** 10.1186/s40560-018-0314-8

**Published:** 2018-07-28

**Authors:** Taiga Itagaki, Yoshitoyo Ueno, Nobuto Nakanishi, Jun Oto

**Affiliations:** 1 Department of Emergency and Critical Care Medicine, Tokushima University Graduate School, 3-18-15 Kuramoto-cho, Tokushima, 770-8503 Japan; 2 Department of Emergency and Disaster Medicine, Tokushima University Hospital, 2-50-1 Kuramoto-cho, Tokushima, 770-8503 Japan

## Abstract

Reverse triggering is respiratory entrainment triggered by the ventilator especially seen among heavily sedated patients. We confirmed reverse triggering induced by auto-triggering in lightly sedated patient through an esophageal pressure monitoring. The reverse triggering frequently caused breath stacking with increased tidal volume. Physicians should be aware, even at an optimal level of sedation, that reverse triggering can develop, possibly caused by auto-triggering.

To the Editor,

Patient-ventilator asynchrony is common and associated with increased duration of mechanical ventilation, ICU length of stay and mortality [1, 2]. Reverse triggering is diaphragmatic muscle contraction induced by passive insufflation of the lungs, especially in deeply sedated patients [3, 4]. Through an esophageal pressure monitoring, we confirmed reverse triggering induced by auto-triggering in lightly sedated acute respiratory distress syndrome patient.

Three days after an emergency operation for bowel perforation (day 0), a 67-year-old man (161 cm, 55 kg) was admitted to the ICU for respiratory failure. Chest radiography showed bilateral diffuse infiltration. Owing to severe hypoxia (PaO_2_/F_I_O_2_ 120 mmHg), mechanical ventilation in pressure assist-control mode was started along with continuous infusion of fentanyl. On day 4, pneumocystis pneumonia was diagnosed. On day 9, to redress oxygenation deficit, PEEP 14 cmH_2_O and F_I_O_2_ 0.8 was required to keep SpO_2_ greater than 92%. On day 11, he scored − 1 on the Richmond agitation–sedation scale. Meanwhile, with the following ventilator settings: inspiratory pressure above PEEP 12 cmH_2_O, PEEP 14 cmH_2_O, inspiratory time 1.0 s, frequency 12 breaths/min and flow trigger sensitivity 3.0 L/min, ventilator graphics showed frequent double cycling interspersed with apparently normally triggered breaths (Fig. 1a). Esophageal pressure monitoring revealed repeated auto-triggering followed by reverse triggered breaths (Fig. 1c). We determined that air leak was causing auto-triggering, which we then effectively prevented by increasing endotracheal tube (ETT) cuff pressure (Fig. 1b). Thereafter, every machine cycle was preceded by neural effort of the patient (Fig. 1d).

**Fig. 1:**
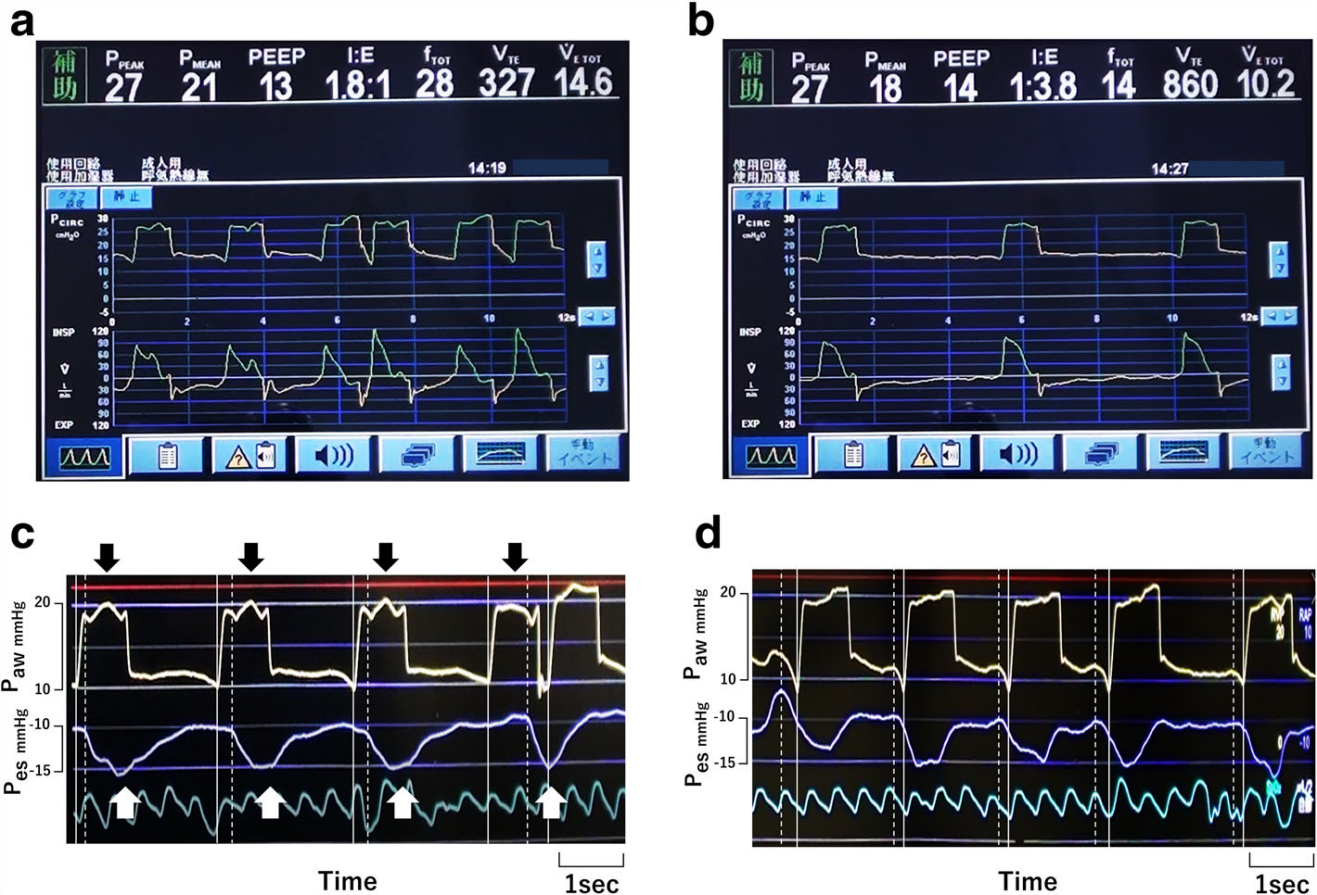
**a**, **b** Airway pressure (Paw, *top*) and flow (*bottom*) waveforms over time during pressure assist-control ventilation. **c**, **d** Paw (*top*) and esophageal pressure (Pes, *bottom*) tracings. Solid lines indicate the start of machine cycles and dotted lines indicate the start of neural efforts. **c** All cycles (black arrows), occurring at 22 breaths/min, more than the set frequency, were auto-triggered rather than time-cycled breaths. White arrows indicate entrained breaths (reverse triggering) triggered by auto-triggered breaths. At the fourth breath, owing to a second machine cycle that was triggered by the entrained breath, “breath stacking” occurred. **d** After preventing auto-triggering by increasing ETT cuff pressure, neural efforts preceded machine cycles while the order of machine cycle and neural effort was reversed in **c**

Auto-triggering is defined as a machine cycle delivered by the ventilator without triggering by the patient. It is observed in patients with air leak, excessive water in the circuit, high trigger sensitivity, or cardiac oscillations [2, 5]. In the case presented above, we confirmed that auto-triggering was the origin of reverse triggering through an esophageal pressure monitoring. We stress the importance of proper ETT cuff management by critical care providers to prevent potentially harmful complication, because auto-triggering can be solved completely once we remove air leak.

Although clinical impact of reverse triggering remains unclear, it is an issue if reverse triggering produces double cycling (breath stacking) with increased tidal volume. Generally, to eliminate breath stacking, setting longer inspiratory time is considered [6]. Even in the case of breath stacking beginning with auto-triggering, this strategy theoretically decrease the occurrence of breath stacking. However, once auto-triggering has been resolved, setting longer inspiratory time may cause significant delayed cycling and ultimately, dynamic hyperinflation of the lung. Physicians should be aware, even at an optimal level of sedation, that reverse triggering can develop, possibly caused by auto-triggering and lead to lung-injurious breath stacking.

## References

[1] Blanch L, Villagra A, Sales B, Montanya J, Lucangelo U, Luján M (2015). Asynchronies during mechanical ventilation are associated with mortality. Intensive Care Med.

[2] Itagaki T, Nishimura M (2017). Patient-ventilator asynchrony during assisted mechanical ventilation. J Jpn Soc Intensive Care Med.

[3] Akoumianaki E, Lyazidi A, Rey N, Matamis D, Perez-Martinez N, Giraud R (2013). Mechanical ventilation-induced reverse-triggered breaths: a frequently unrecognized form of neuromechanical coupling. Chest.

[4] Murias G, de Haro C, Blanch L (2016). Does this ventilated patient have asynchronies? Recognizing reverse triggering and entrainment at the bedside. Intensive Care Med.

[5] Tobin MJ, Jubran A, Laghi F (2001). Patient-ventilator interaction. Am J Respir Crit Care Med.

[6] Chanques G, Kress JP, Pohlman A, Patel S, Poston J, Jaber S, Hall JB (2013). Impact of ventilator adjustment and sedation-analgesia practices on severe asynchrony in patients ventilated in assist-control mode. Crit Care Med.

